# Tailoring Light-Weight Aggregates for Concrete 3D Printing Applications

**DOI:** 10.3390/ma16072822

**Published:** 2023-04-01

**Authors:** Yi Wei Daniel Tay, Ming Jen Tan, Teck Neng Wong

**Affiliations:** Singapore Centre for 3D Printing, School of Mechanical & Aerospace Engineering, Nanyang Technological University, Singapore 639798, Singapore

**Keywords:** packing factor, additive manufacturing, 3D concrete printing, lightweight materials, rheology

## Abstract

Concrete 3D printing is a sustainable solution for manufacturing efficient designs and creating less waste, and selecting the optimal materials to use can amplify the advantages of this technology. In this study, we explore printing lightweight concrete by replacing normal weight aggregate with lightweight aggregates such as cenospheres, perlite, and foam beads. We adopt a systematic approach to investigate mixtures using different formulation methods such as the specific gravity and packing factor methods to improve the printing and mechanical performances of the mixtures. The rheological results showed significant improvement in the flow characteristics of the different mixtures using both the specific gravity method and the packing factor method to formulate the mixtures. Furthermore, a statistical tool was used to achieve optimal performance of the mixtures in terms of high specific compressive strength, high flow characteristics, and good shape retention capability by maximizing the specific compressive strength ratio, slump flow, and the static yield stress, while minimizing the slump, dynamic yield stress, and plastic viscosity. With the above design objectives, the optimal percentages of the aggregate replacements (cenosphere, perlite, and EPS foam beads) were 42%, 68%, and 44%, respectively. Finally, the optimized results also showed that the mixture with cenosphere aggregate replacement had the highest specific strength.

## 1. Introduction

The use of lightweight concrete offers various economic and energy conservation advantages [1]. One of the primary benefits of lightweight concrete is its ability to reduce the dead load of structures, enabling architects to decrease the size of columns and other load-bearing elements [1]. There are several methods for producing lightweight concrete. Due to the higher porosity of lightweight concrete, it also provides good insulation properties, including heat preservation, heat insulation, and sound absorption [2]. The mechanical strength of concrete and mortar is closely related to the density and the volume fraction of the aggregates [3]. Lightweight concrete can be created by aeration, by removing the fine aggregates in concrete, and by using lightweight aggregates [4,5,6].

**Figure 1:**
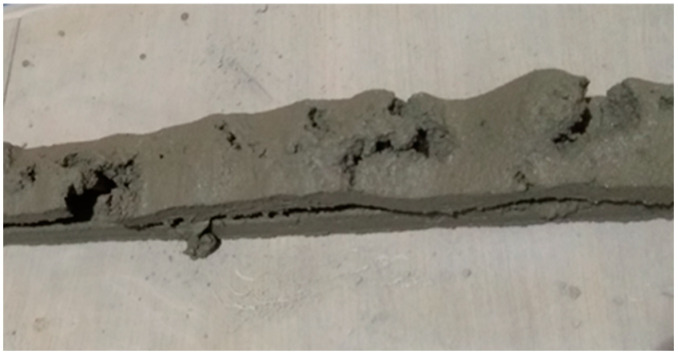
Defect in the printed foam concrete.

Aerated concrete is composed of mortar and sand with entrapped air bubbles that are created through chemical or mechanical means [7]. Chemical methods for preparing aerated concrete involve using a gas-forming agent during the plastic or liquid state, which results in an increase in volume as the gas escapes and leaves behind a porous structure [8]. The most common material used as a gas-forming agent is aluminium powder; the efficiency of the gas-foaming process is influenced by the fineness and purity of the aluminium powder, as well as the method used to prevent the gas from escaping before the mortar hardens. Mechanically prepared aerated concrete, also known as foam concrete, is made using either the pre-foaming method or the mixed foaming method [9]. In the pre-foaming method, the foam and mortar are prepared separately before being mixed together to create the foam concrete, whereas in the mixed foaming method, all the materials, including the surfactant or foaming agent, are mixed together from the start. The density of the foam concrete is controlled by the amount of foam mixture or surfactants added, and striking a balance between strength and density is necessary to maximize strength while keeping the weight as low as possible [10]. Aerated lightweight concrete is a high thermal-insulating construction material that is used for both interior and exterior construction, and one of its advantages is the ease of installation due to the use of standard power tools. However, we attempted to create printable foam concrete, but due to an excessive amount of air in the foam concrete, printing was not possible. The air within the foam concrete caused a burst of air to explode as the material exited the nozzle during extrusion, resulting in an inconsistent flow as it extruded (See Figure 1). Therefore, this method was rejected.

The lightweight properties of “no-fine” concrete, which is also known as pervious concrete, has been attributed to the absence of fine aggregates, resulting in a high percentage of interconnected voids [11]. However, this porosity also allows water to penetrate the material matrix. The aggregate size typically ranges from 10 to 20 mm [4]. Al-khalaf and Yousif [12] noted that there was a lower limit to the density that no-fine concrete could achieve without losing its cellular nature, and this balance between density and cellular nature depended on the intended application as either a structural or insulating material. While no-fine concrete has many applications, it is commonly used for pavement and roads due to its high permeability that prevents skidding [13]. For a material to be pumpable, it must be able to flow smoothly through a nozzle without clogging and the maximum particle size recommended by our pump supplier is 2 mm. Therefore, printing of no-fine concrete is not possible since the average size of aggregates used is 10–20 mm.

Hence, in this study, we investigated the last method of creating printable lightweight concrete, which is to incorporate light weight aggregates. Replacing normal weight aggregates with lightweight aggregates is the most common way of creating lightweight concrete; it reduces the density of the concrete and retains a certain strength of the concrete [6]. The use of lightweight aggregates is more complex than that of normal weight aggregates, due to the varying absorptivity of water and specific gravity. Lightweight aggregates have a lower bulk specific gravity due to their ability to entrap air in the cellular structure. Numerous studies in the literature have proposed using the specific gravity factor to formulate concrete with lightweight aggregates [3,14], since it enables the quantities of lightweight aggregates to be determined and controlled.

Concrete 3D printing is a sustainable solution because of its ability to manufacture optimized designs and to create less waste [15]. Nguyen et al. [16] and Barjuei et al. [17] optimized the 3D printing process using a machine learning and monitoring system. They streamlined the process to reduce the time and effort, while also improving printing accuracy. While their approach has improved the quality and sustainability of the manufacturing process, more needs to be done to improve the materials aspect of the process. The correct use of sustainable materials and the selection of a sustainable complex architectural design can amplify the advantages of this technology [18]. With a printable mixture formulated previously with natural river sand, in this paper, we present a systematic approach to investigate the effects of replacing natural river sand with different lightweight aggregates. The amounts of the lightweight aggregates, i.e., cenospheres, perlite, and styrofoam beads were varied to evaluate the feasibility and performance of the materials in terms of rheological properties and mechanical properties. A reference material with natural river sand was used as the control for comparison with the performances of the printable materials. Several tests were performed to evaluate the materials’ performances for 3D printing concrete applications, and therefore, to formulate the optimal mixture for each of the lightweight aggregate replacements. The subsequent sections present the results obtained.

## 2. Materials and Methods

### 2.1. Material Preparation

The raw materials used in this study were ordinary Portland cement (OPC, ASTM Type 1, Grade 42.5), silica fume (Undensified, Elkem, Oslo, Norway), fly ash (Class F), superplasticizer, river sand (specific gravity of 2.741), cenospheres (specific gravity of 0.72), perlite (specific gravity of 0.746), and foam beads (specific gravity of 0.013). The plasticizer was added to improve the flowability of the mixture, since it acts as a dispersant that reduces the surface tension between the cement particles and the mixing water, which allows the particles to move more freely and remain suspended in the mixture. Therefore, less water was added which improved the strength and durability of the concrete.

**Figure 2:**
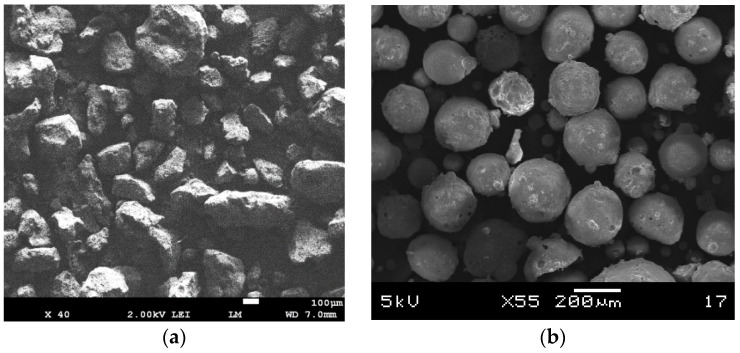

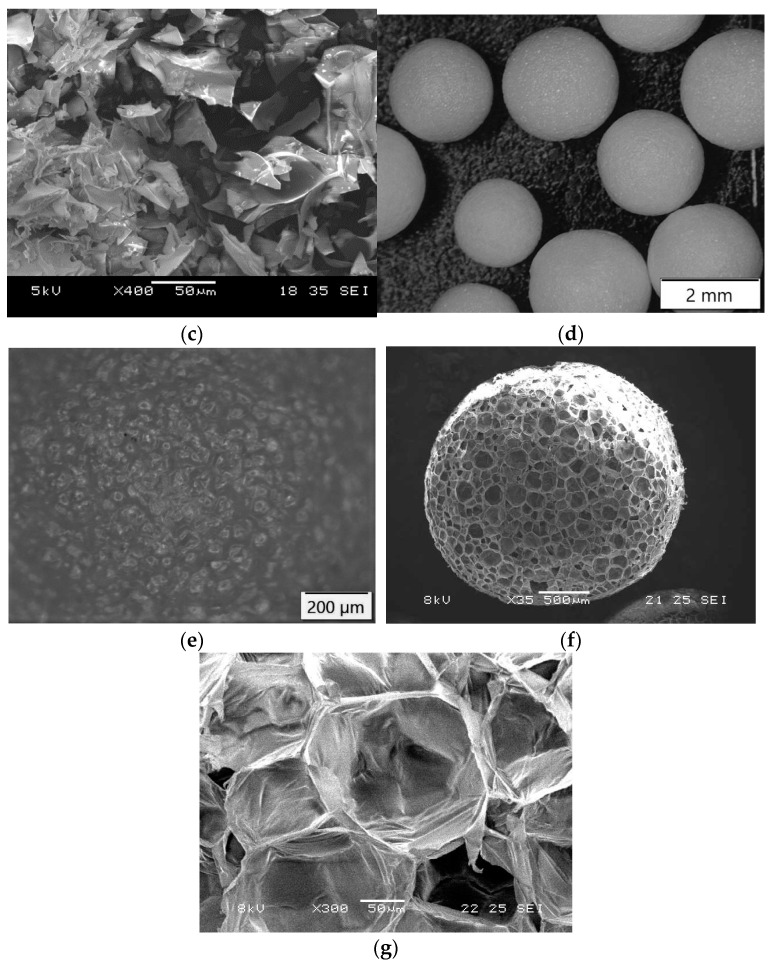
Microscope images of: (**a**) River sand; (**b**) cenospheres; (**c**) perlite; (**d**) foam beams; (**e**) the surface of a foam bead consisting of smaller encapsulated bubbles; (**f**) a cross-sectional image of a single foam bead; (**g**) a cross-sectional close-up image of a foam bead showing the enclosed bubbles.

The specific gravity and absorption of the aggregates were tested according to ASTM C128 [19]. These tests were independent of the specific gravity or packing factor methods described in Section 3.1 and Section 3.2. A water absorption test was conducted to determine the extent to which the aggregates absorbed free water during the mixing process. The absorption of such water could serve as a lubricant for the aggregates. The tests were repeated at least three times to obtain an average value. While there may be the presence of broken or cracked cenospheres in the mixture (see Figure 2b), a small quantity does not significantly affect the results. It is difficult to mitigate these broken cenospheres which represent a characteristic of the material’s behavior.

Microscopic images of river sand, cenospheres, perlite, and foam beads are shown in Figure 2. The river sand aggregate partiles are mostly edged and consist of different sizes and shapes. The perlite aggregate particles have sharper edges compared to those of the sand particles and look like edged flakes that are pieced together. In contrast, cenosphere and foam bead particles have a more uniform size and are spherical in shape. The cenosphere aggregate particles consist of an enclosed shell which traps air within the shell. Some reports have shown that the thicknesses of the cenosphere shells are approximately from 3% to 11% of the diameter of the cenospheres [20,21]. Similarly, foam bead particles also trap air within their spherical bodies. As seen in Figure 2e–g, the foam bead particles consists of smaller encapsulated bubbles, which have a better capability of trapping air.

To ensure that the different samples prepared with different lightweight materials were comparable, the cementitious material was kept constant, which consisted of cement, fly ash, and silica fume. Only the aggregate content was varied. In this study, a code was assigned to the materials investigated. The first letter of a code represents the lightweight aggregates used, the second and third letters of a code represent the formulation method, and the remaining digits indicate the amount of material replaced. For example, when perlite with a replacement volume of 20% was replaced using the specific gravity (SG) method, the code name is P-SG20. Information on the cementitious material mixture design used in this study cannot be disclosed due to the non-disclosure agreement with the collaborators.

The mixtures were delivered by means of pumping through a hose with a diameter of 2.54 mm and the maximum particle size allowed by the pump specification was 2 mm. A Hobart mixer was used to prepare all mixtures. Dry components were dry-mixed at 59 rpm for 5 min, and then water was added to the mixture and mixed at a slow speed (59 rpm) for 1 min which was followed by 7 min of high-speed mixing (198 rpm).

### 2.2. Testing Protocols

#### 2.2.1. Slump and Flow Table Tests

Slump and flow table tests are field-friendly tests that are used to determine the workability of a material. A conical mold was used for this experiment in accordance with ASTM C230 [22]. The slump test procedure began by filling half of the mold with mortar. Then, the mold was tamped 20 times to compact the mortar uniformly. More mortar was added to the brim of the mold and tamped 20 more times to compact the mortar. Excess material was removed by using the edge of a trowel with a sawing motion.

The slump value is the difference between the top of the mortar and the top of the mold after the mold is removed. The flow table test continued from the slump test where the flow table was dropped 25 times and the flow diameter of the mortar was recorded which was called the slump flow value. This test has been previously established in the literature [23].

#### 2.2.2. Rheological Testing

**Figure 3:**
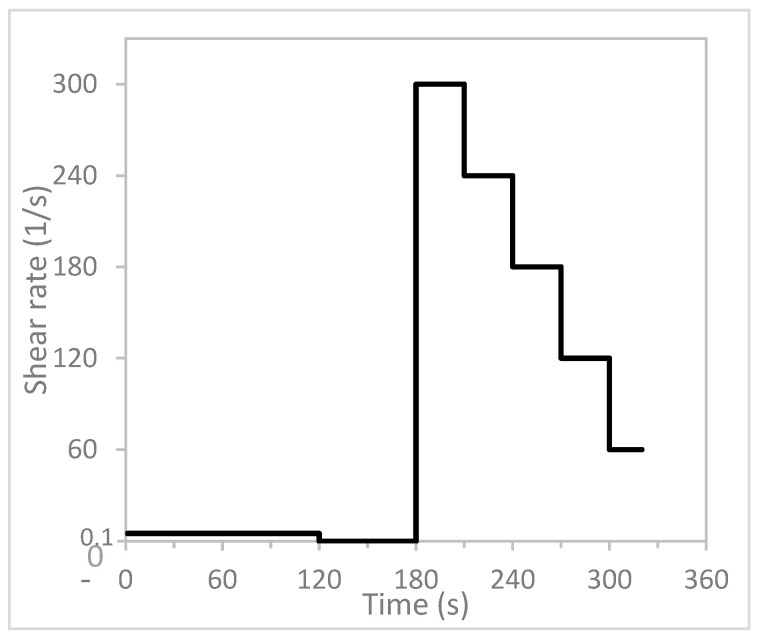
Rheological protocol.

A rheometer (Anton Paar MCR102, Graz, Austria) was used to characterize the rheological properties of the materials. The rheometer was equipped with a four-blade measuring stirrer and a cup. The cup had a modular insert cage with serrations to prevent wall slippages. Figure 3 shows the rheological protocol used for this study. The protocol consists of three parts. The first part of the protocol involves rotation at a shear rate of 0.1 s^−1^ for 120 s to capture the material’s static yield stress at a low shear rate. This low shear rate allows the material’s shear stress to be captured as it gradually increases. As the shear rate continues, the shear stress reaches its peak. The shear stress value is captured as its static yield stress when the material starts to deform in the measuring cup [24].

The second part of the protocol, from 120 s to 180 s, is the time period during which the material rests. The third part of the protocol involves measuring the material’s plastic viscosity and dynamic yield stress, which involves reducing the shear rate in a stepwise manner and measuring in 30 s intervals.

#### 2.2.3. Mechanical Strength Testing

**Figure 4:**
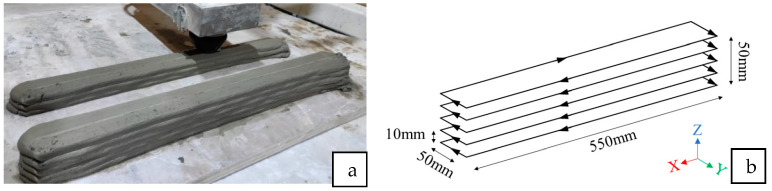
(**a**) Printed block for mechanical analysis; (**b**) print path of the printed block with dimensions.

Mechanical strength was used as a performance parameter to determine the optimal content of the aggregate replacements in the printable mixtures. This test aimed to obtain the strength characteristics of the different aggregate replacements; therefore, the compressive test was performed on cast samples. There are numerous studies in the literature that have shown that cast samples have higher strength as compared to printed samples [25,26]. This is mainly due to the voids in between the filaments caused by the printing parameters, printing path, and nozzle geometry. The mechanical strength test was carried out in compliance with the ASTM C109 standard [27]. The cast samples for the compressive test were created as 50 mm length cubes. The printed samples were also cut to the same dimensions using a diamond cutter (Secotom-60, Cleveland, OH, USA). The printed samples were tested only in the Z-direction, as illustrated in Figure 4b. The cast and printed samples were both left in the mold and on the print table, respectively, for 24 h, and covered with plastic wrap. Then, the samples were immersed in tap water for curing until the 28th day prior to testing.

### 2.3. Printing Parameters

A three-axis gantry printer and a progressive cavity pump were used for the printer setup in this study. The material is delivered from the hopper of the pump to the nozzle by a three-meter hose. The extrusion of material is caused by the rotation of the single helix rotor inside the stationary stator. As the rotor turns, the helix moves along the length of the stator, creating a series of expanding and contracting cavities between the two components. As the cavities expand, they create a low-pressure zone that draws fluid into the pump through the inlet port. As the cavities contract, they push the fluid towards the outlet port. This moves the material through the pump and the hose in a smooth and continuous flow without pulsation.

Table 1 shows the printing parameters used in this study. The printing speed of the gantry was fixed at 100 mm/s for all mixtures. After the printing process, the printed blocks (see Figure 4) were cut into smaller cubes of 50 mm and the procedure for testing their mechanical strength was in accordance with the ASTM C109 standard [27].

All specimens had a solidity ratio (*SR*) in the range from 1.1 to 1.2. The *SR* value was used to determine the consistency of the printed filament based on the printing parameters [28]. Two different printing parameters can have the same *SR* which means the amount of material deposited per unit length is the same. *SR* is defined by Equation (1):

(1) SR=1000Q  An×Vn

where $A_{n}$ is the area of the nozzle orifice, $V_{n}$ is the travel speed, and *Q* is the flow rate of the material.

## 3. Results

The different mixtures’ properties were characterized by using slump and flow table tests, rheological measurement tests, and mechanical strength tests. Each test determines a material’s performance during printing and should be optimized. According to Tay et al. [23], slump value and flow table value correlate well with the pumpability and buildability performances, respectively, of a material during printing.

### 3.1. Specific Gravity Method

To obtain the optimal mixture design, different amounts of the lightweight aggregate replacements must be tested to map out the characteristics of the materials’ performances. Lightweight aggregates have different densities compared to river sand aggregate. If direct weight replacement is carried out without considering density, more lightweight aggregate is added to a mixture in terms of volume. This causes the mixture to be unworkable since more aggregate surface area is introduced, reducing the amount of excess paste [29]. Therefore, the specific gravity (SG) of the lightweight aggregates was used to determine the amount of replacement. This allowed the same amount of aggregate volume to be replaced; 20%, 60%, and 100% volumes of the river sand were replaced with the same volume of the respective different lightweight aggregates.

#### 3.1.1. Slump and Flow Table Test Results

**Figure 5:**
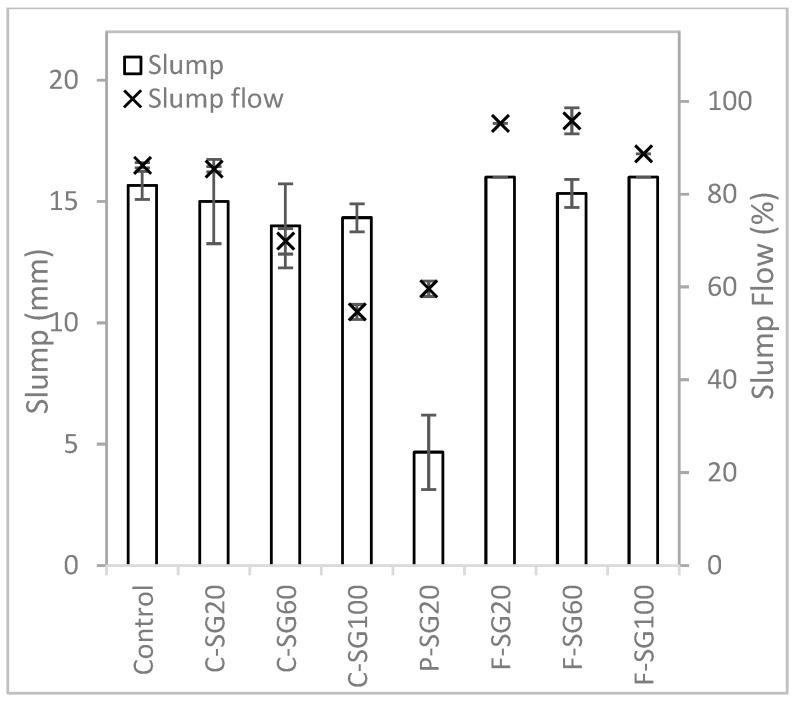
Slump and slump flow results of mixtures prepared with the specific gravity method.

The results of the slump and flow table tests are presented in Figure 5. It can be observed that the mixtures containing cenosphere aggregate as a replacement for river sand have lower values for slump and slump flow compared to the control mixture. The slump flow decreases with increasing cenosphere content. In contrast, the mixtures with foam bead aggregate replacement exhibit different behaviors compared to those of the other two aggregates. The slump decreased when the foam bead aggregate replacement was increased from 20% to 60%, but increased when the foam bead aggregate replacement was increased from 60% to 100%. Moreover, the slump flow increased when the foam bead aggregate replacement was increased from 20% to 60% and decreased when the foam bed replacement was increased from 60% to 100%. Mixtures with perlite aggregate replacement were stiff, as depicted in Figure 5. The P-SG60 and P-SG100 mixtures were too dry to be analyzed, likely due to perlite’s high water absorption, as indicated in Table 2. Because mixtures with perlite aggregate replacement were too stiff to be analyzed, the results were unacceptable for comparison with the other mixtures, and thus, were not included in subsequent analyses in Section 3.1.2 and Section 3.1.3.

#### 3.1.2. Rheological Analysis

**Figure 6:**
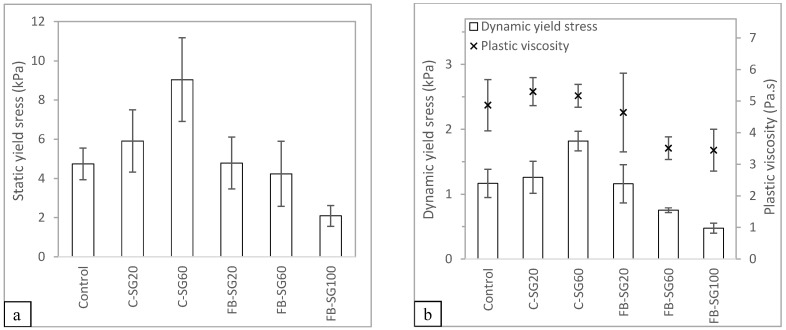
(**a**) Static yield stress results and (**b**) dynamic yield stress results of mixtures prepared with the specific gravity method.

The rheological measurement results obtained for the different test mixtures are shown in Figure 6. It should be noted that the rheological result for C-SG100 was inconclusive, as the rheometer reached its maximum torque before the measurement could be captured. Nonetheless, the material appears to be workable, as evidenced by the results of the slump and slump flow tests. The stiffness of the material, however, was much higher than what the rheometer could measure. Figure 6a depicts the static yield stress results of the different test mixtures. Notably, replacing river sand with cenosphere aggregate increases the static yield stress of the mixtures, while mixtures containing foam bead aggregate replacement exhibit significantly lower static yield stress.

Figure 6b illustrates the dynamic yield stress results of the various mixtures. It is observed that, on the one hand, the mixtures containing cenosphere aggregate replacement exhibit increasing dynamic yield strength and decreasing plastic viscosity with a higher proportion of cenosphere aggregate replacment in the mixture. On the other hand, the foam bead aggregate replacement displays a different behavior compared to that of the cenosphere aggregate repalcement. In the mixtures containing foam bead aggregate replacement, both the dynamic yield stress and plastic viscosity decrease with an increasing amount of foam bead aggregate replacement.

Cenosphere aggregate replacement, compared to river sand, has more smaller-sized particles. Despite the replacement volume being the same as river sand, the larger surface areas of the smaller cenosphere particles reduce the amount of paste between the concrete, thereby, increasing the yield stress and viscosity [30]. According to Hoorndhad [31], the distance between the aggregate particles affects the yield stress and viscosity of the mixtures. This is due to the interaction between the aggregate particles during flow. On the one hand, the high yield stress and plastic viscosity of the mixtures with cenosphere aggregate replacement show that the mixtures require a higher pumping pressure to achieve the same flow rate as the control mixture. On the other hand, the lower yield stress and plastic viscosity of the foam bead aggregate replacement would be easier to pump through during the pumping process, however, such fluid-like characteristics might not be ideal for printing as it may collapse under its weight.

#### 3.1.3. Mechanical Test Results

**Figure 7:**
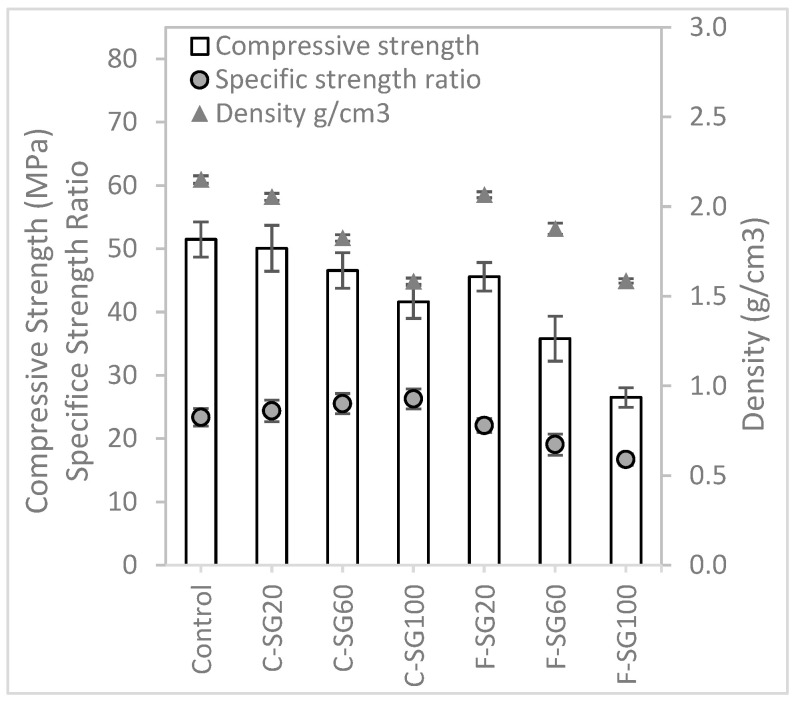
Compressive test results of mixtures prepared with the specific gravity method.

Figure 7 illustrates the compressive and flexural strength of the different mixtures. As expected, test mixtures with lightweight aggregates exhibit lower compressive strength compared to the control mixture. The strengths of these lightweight aggregate samples is largely dependent on the strength of the aggregates. For lightweight concrete, the weakest link is often the aggregates rather than the interfacial zone [32]. The mixtures with cenosphere or foam bead aggregate replacments both display similar trends, with compressive strength decreasing as the amount of lightweight aggregate used increases. Notably, the mixtures with cenosphere aggregate replacement exhibit higher compressive strength than those with foam bead aggregate replacement.

In this study, specific compressive strength and specific flexural strength are defined as the ratios of the respective strength of concrete and the density of the material. It was observed that the mixtures with cenosphere aggregate replacement exhibited an increasing trend in specific strength as the cenosphere aggregate replacement content increased. For the mixtures that had 60% and 100% cenosphere aggregate replacement, they had higher specific strength as compared to the control mixture which had no lightweight aggregate in the mixture. This indicates that cenosphere aggregate replacement has a positive impact on the specific strength of concrete. Conversely, mixtures with foam bead aggregate replacement exhibit a reduction in specific strength, suggesting that foam bead aggregate replacement has a negative impact on the specific strength ratio.

### 3.2. Packing Factor Method

A more refined methodology was performed to improve the performances of the mixture design of different aggregate replacements. In the previous batch of testing using the specific gravity method, the different behaviors of the various lightweight aggregate replacements led to variations in the stiffness of the concrete mixtures. We concluded that different packing densities and absorptivity of the aggregates drastically affected the characteristics of the concrete mixture. This was observed from a previous batch of test samples where mixtures with perlite as the lightweight aggregate became too dry to conduct any testing on the samples. Using the specific gravity method as presented in Section 3.1, mixtures with perlite aggregate replacement were too stiff to be used for testing and the result would be inconclusive. For a more conclusive result, and for perlite to be compared with other lightweight material, the packing factor (PF) method and water absorption were used as key calibrators for the new set of experiments.

The rheology of concrete can be significantly affected by the surface area of aggregate particles. Generally, as the surface area increases, the flowability decreases due to a decrease in the thickness of water film coating solid particles. When the surface area is larger, the water film thickness decreases, leading to lower flowability with the same amount of excess paste, and vice versa. In this study, to ensure sufficient paste material is present to lubricate between the aggregate particles, the packing factor of the aggregates is taken into consideration. The packing factor is a measure of how efficiently aggregate particles are packed together in a given volume of space. Aggregates with higher surface areas tend to have lower packing factors because the irregular shape of the particles makes them harder to pack together efficiently. Conversely, aggregates with higher packing factors tend to have lower surface areas because their more regular shape allows for tighter packing. On the one hand, the packing factor method considers the aggregate shape and size, as well as the interaction between the aggregate particles. On the other hand, the surface area method only considers the amount of surface area present and does not consider the interaction between the particles. A mixture that considers only surface area may require a higher amount of paste, resulting in higher flowability. Therefore, when formulating a mixture, it is more critical to consider the aggregate packing factor than just the surface area.

Interstitial spaces and voids between aggregate particles in a compact envelope are unavoidable. Reducing the voids also reduces the amount of water and cement paste required to bind the aggregate particles, since the volume of the space present in a dense pack mixture design is less. The packing factor method was adopted from Hoornahad [31] and Raj et al. [33]. This method was utilized to improve the material consistency of mixtures with lightweight aggregate content. The detailed protocol for testing is recorded in the existing literature [31]. The packing factor ***ς*** can be expressed by Equation (2):

(2) $$\varsigma =\frac{V_{a}}{V_{b}}\leq 1$$

where V_a_ is the specific volume of the aggregate and V_b_ is the bulk volume of the aggregate which represent an aggregate skeleton in a compacted state. The packing density of an aggregate depends on the particle size distribution, shape characteristics of the aggregate particles, and packing method. Equation (3) can be used to determine the amount of the lightweight aggregate in the mixture by weight:

(3) (ςLςs)×ρL×VL=WL

where *ς_L_* is the respective lightweight aggregate packing factor, ςs is the sand packing factor, ρ_L_ is the respective lightweight aggregate density, V_L_ is the respective lightweight aggregate volume, and W_L_ is the respective lightweight aggregate weight in the mixture. Excess water was added based on the water absorption capabilities of the aggregates as presented in Table 2. Table 3 shows the packing factor of the respective aggregates that were used in the calculations.

#### 3.2.1. Slump and Flow Table Test Results

**Figure 8:**
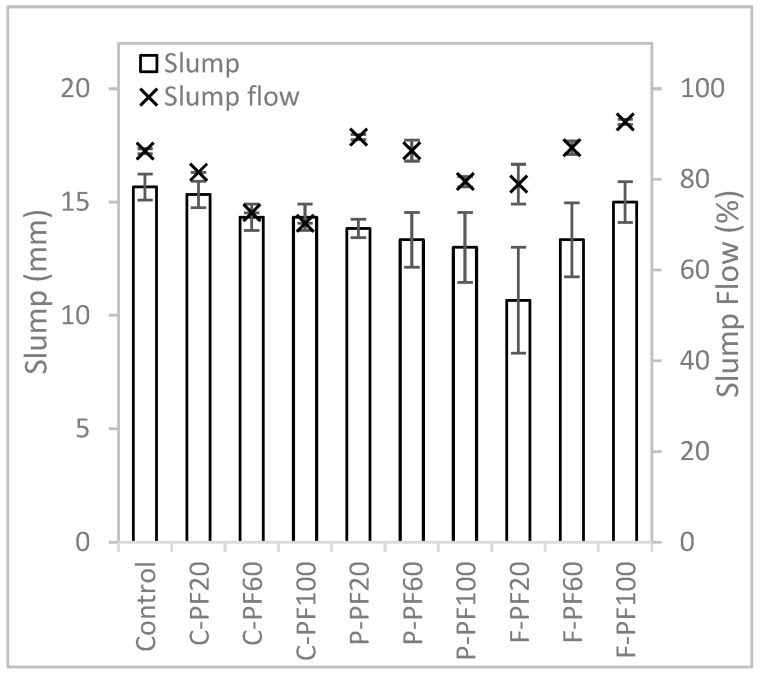
Slump and slump flow results of mixtures prepared with the packing factor method.

It was observed that the mixtures with perlite aggregate replacement had better flow capability and were more workable than the previous batch. This is due to less perlite in the matrix and an increase in water content when the packing factor and absorptivity of the aggregates are considered in the design process of the mixture formulation method. As shown in Figure 8, mixtures with cenospheres and perlite aggregate replacements show a similar trend, as the aggregate content replacement increase, the mixture reduces in slump and slump flow. This means that the mixture stiffness increases as more river sand aggregate is replaced with perlite or cenosphere aggregates. The mixtures with perlite aggregate replacement generally have a higher slump flow and lower slump value as compared to the mixtures with cenosphere aggregate replacement. This shows that perlite aggregate replacement has better flow capability and the cenosphere aggregate replacement has better shape retention capability. Mixtures with foam bead aggregate replacement behave differently compared to the other two aggregates. Slump and slump flow increase as more river sand aggregate is replaced with foam bead aggregate, due to the smooth surface and hydrophobic feature and low density of the foam beads, which is in good agreement with the existing literature [34].

When the packing factor method is considered in the mixture formulation method, most of the mixtures have a lower amount of aggregates to be replaced. Since the packing factor for most of the lightweight aggregate is less than the river sand, as shown in Table 3, the amount of aggregate added to the mixture is reduced as compared to the mixtures formulated using the specific gravity method. A comparison of the results in Figure 8 with Figure 5 shows that most lightweight aggregate mixtures have a lower slump value and higher slump flow value.

#### 3.2.2. Rheological Analysis

**Figure 9:**
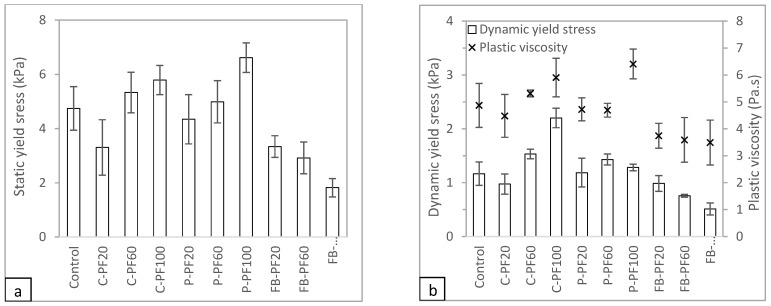
(**a**) Static yield stress and (**b**) dynamic yield stress and plastic viscosity results of mixtures prepared with the packing factor method.

The results for the static yield stress of the test amples were collated and are presented in Figure 9a. As observed in the slump and slump flow results, the static yield stress of the mixtures with cenosphere and perlite aggregate replacements increases with an increase in the percentage of replacement. Moreover, the mixtures with cenosphere and perlite aggregate replacements have higher static yield stress as compared to the control sample. However, the behavior of the samples with foam bead aggregate replacement is different from those of the other two aggregate groups. The static yield stress of the mixture decreases as the percentage of foam bead aggregate replacement increases in the sample.

The results shown in Figure 9b summarize the dynamic yield stress and plastic viscosity of the different test samples. All three groups of lightweight aggregate replacement have very different behaviors. For the mixtures with cenosphere aggregate replacement, on the one hand, the dynamic yield stress increases as the replacement percentage increases. On the other hand, plastic viscosity reduces with an increase in the percentage of cenosphere aggregate replacement. Cenosphere particles are more regular and rounder in shape as compared to river sand particles which have a more jagged and irregular shape. Thus, allowing cenosphere particles to slide among each other easily reduces the plastic viscosity as the replacement percentage increases. For the mixtures with perlite aggregate replacement, the dynamic yield stress increases when the aggregate replacement is increased from 20% to 60% and subsequently reduces. Conversely, the plastic viscosity behavior for the mixtures with perlite aggregate replacement reduces from 20% to 60% and increases subsequently. For the mixtures with foam bead aggregate replacement, the dynamic yield stress and plastic viscosity decrease with a higher amount of lightweight aggregate replacement.

#### 3.2.3. Mechanical Test Results

**Figure 10:**
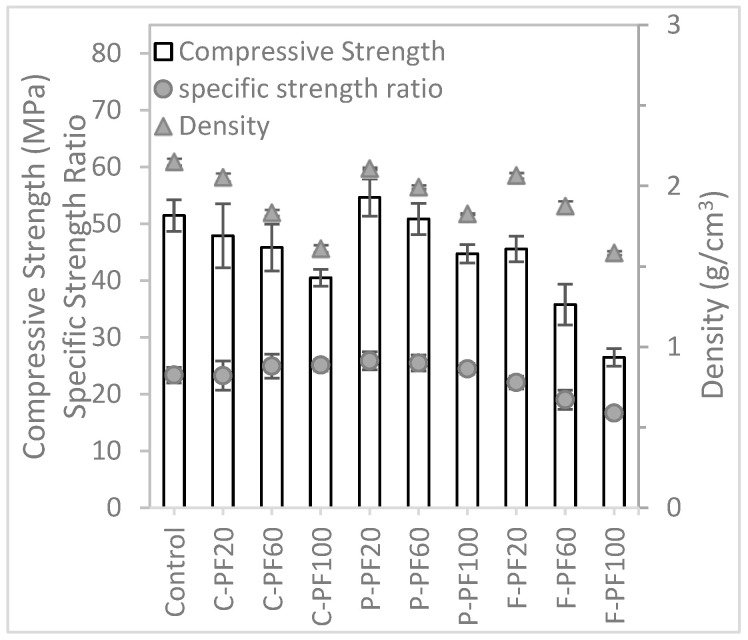
Compressive strength results of mixtures prepared with the packing factor method.

Figure 10 shows the compressive and flexural strength of the mixtures formulated with the packing factor method. From the results obtained, the trend of the compressive strength for all the mixtures with lightweight aggregate replacement are similar, i.e., as more lightweight aggregates replace river sand aggregates, the strength of the mixture reduces. However, perlite aggregate replacement seems to perform better than the samples with cenosphere and foam bead aggregate replacements. Additionally, on the one hand, the specific compressive strength for the mixtures with cenosphere aggregate replacement increases as the aggregate replacement increases. On the other hand, mixtures with perlite and foam bead aggregate replacements experience a reduction in the specific compressive strength as the replacement content increases.

### 3.3. Optimization and Validation of the Results

Optimization was carried out with a statistical tool (Minitab, 17.1.0) with the result obtained described in Section 3.2. The response surface design methodology allowed the response surface to be mapped according to the region of interest that was defined, which can determine the optimal setting for each factor. In the statistical tool, the performance of a mixture was optimized, on the one hand, by maximizing the specific compressive strength ratio, the slump flow value, and the static yield stress. On the other hand, the slump value, dynamic yield stress, and plastic viscosity were minimized to ensure a mixture had a high specific compressive strength, high flow characteristic, and good shape retention capability. With the above design objectives, the optimal percentages of cenosphere, perlite, and EPS foam bead aggregate replacements are 42%, 68%, and 44%, respectively.

#### 3.3.1. Mechanical Test Results

**Figure 11:**
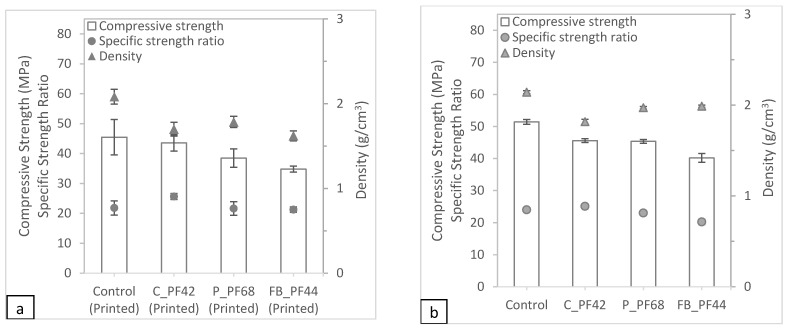
Compressive test results for the optimized (**a**) printed and (**b**) cast samples.

The different mixtures such as the control sample, 42% cenosphere aggregate replacement, 68% perlite aggregate replacement, and 44% foam bead aggregate replacement were printed for further analysis. The compressive test results are shown in Figure 11. While the compressive strength of the control samples may be the highest, the samples with cenosphere aggregate replacement have the best performances in terms of specific compressive strength. In general, the printed samples have lower compressive strength compared to the cast samples, which coincides with the existing literature [35]. In general, it can be observed that the printed samples have lower density and lower compressive strength compared to the cast samples. This lower density leads to some of the samples (samples with cenosphere and foam bead aggregate replacements) having a higher specific strength ratio, even though the compressive strength is lower.

#### 3.3.2. Microscopic Analysis

**Figure 12:**
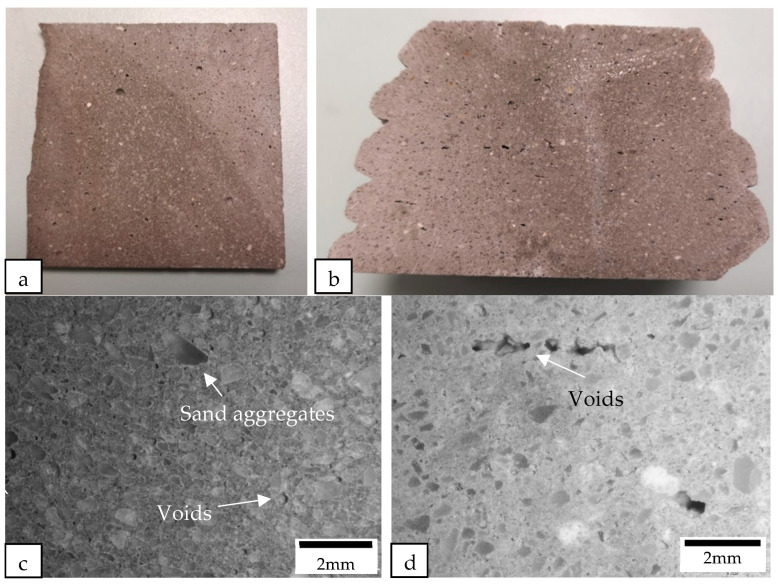
Cross-sectional images of (**a**) the cast control sample and (**b**) the printed control sample were obtained using a camera, while the macrostructure of the cross sections of (**c**) the cast control sample and (**d**) the printed control sample were examined under a microscope.

**Figure 13:**
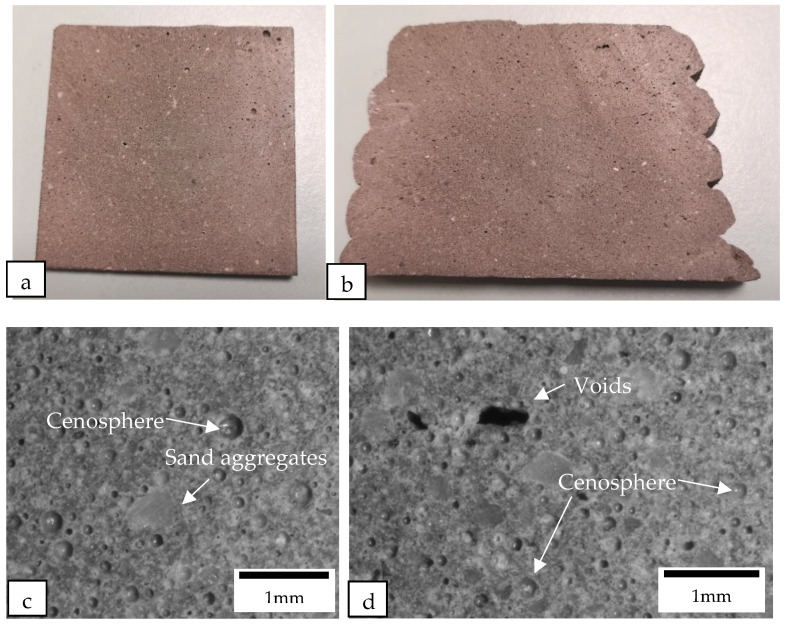
Cross-sectional images of (**a**) the cast and (**b**) the printed samples containing cenosphere aggregate replacement were obtained using a camera, while the macrostructure of the cross sections of (**c**) the cast and (**d**) the printed samples containing cenosphere aggregate replacement were examined under a microscope.

**Figure 14:**
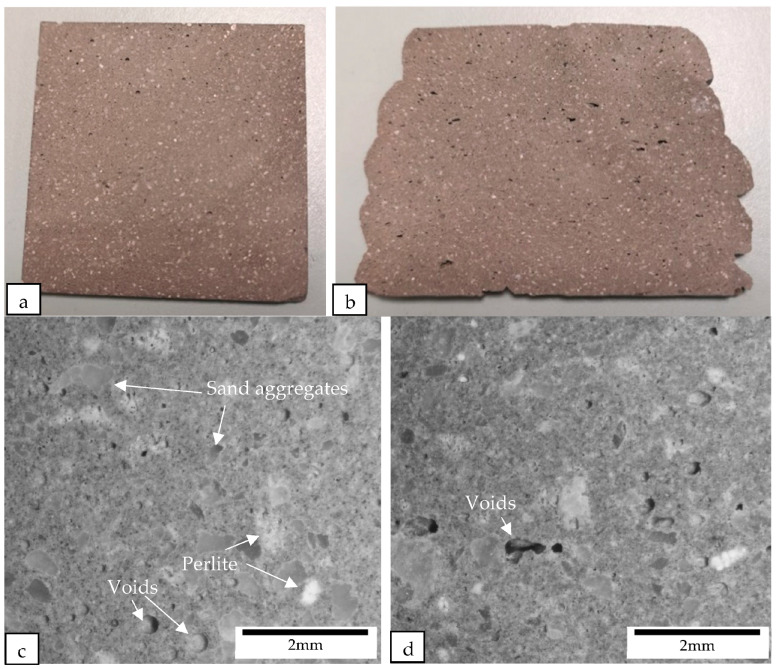
Cross-sectional images of (**a**) the cast and (**b**) the printed samples containing perlite aggregate replacement were obtained using a camera, while the macrostructure of the cross sections of (**c**) the cast and (**d**) the printed samples containing perlite aggregate replacement were examined under a microscope.

**Figure 15:**
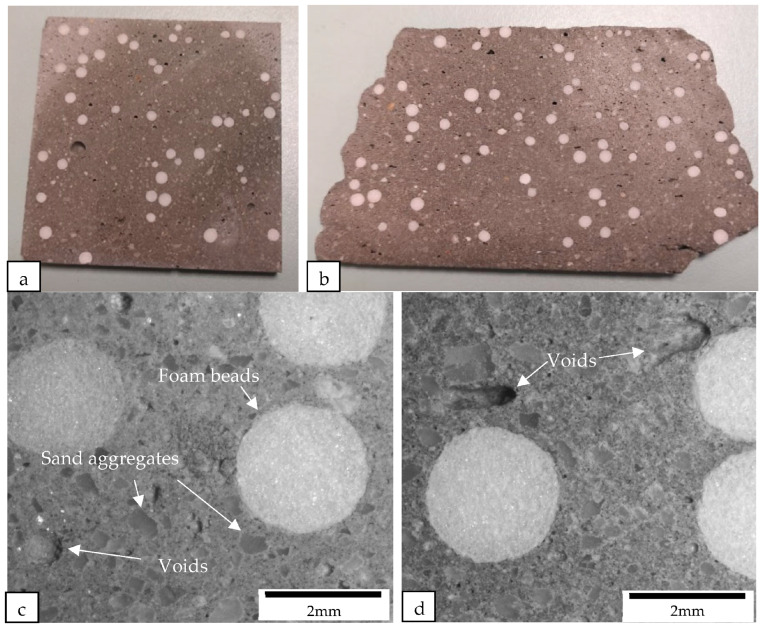
Cross-sectional images of (**a**) the cast and (**b**) the printed samples containing foam bead aggregate replacement were obtained using a camera, while the macrostructure of the cross sections of (**c**) the cast and (**d**) the printed samples containing foam bead aggregate replacement were examined under a microscope.

The samples were cut perpendicular to the printing direction of the filament using a diamond cutter. After cutting, the macrostructures of the samples were examined under a microscope (see Figure 12, Figure 13, Figure 14 and Figure 15). Figure 12 shows the macrostructures of the cast and printed control samples. It can be observed that the material has a slump on the bottom layers, as shown in Figure 12b. The large width of the bottom layer shows that it does not have sufficient strength to support the previous layer. As mentioned by Tay et al. [23], printing parameters also play a role to mitigate slumping of the bottom layers. In order to reduce slumping, the nozzle travel speed needs to be reduced to match the stiffening rate of the material. Figure 12c,d show the macrostructures of the cast and printed control samples, respectively; there are more voids in the printed sample as compared with the cast sample. In addition, as shown in Figure 12d, the voids in the printed sample are generally flattened as compared to the cast sample. These flattened voids were the effects of printing. The extrusion of the concrete filament caused the voids to be compressed in the direction of the nozzle. These flat voids can be observed in all the printed samples (see Figure 12d, Figure 13d, Figure 14d and Figure 15d). These flattened voids may have caused the anisotropic properties in the printed samples, as mentioned by Suvash et al. [36].

Figure 13 shows the cast and printed samples containing cenosphere aggregate replacement. There are significantly more circular pores in both the printed and the cast samples containing cenosphere aggregate replacement compared to the control samples. Most of the circular pores were created due to the round shape of the cenosphere aggregate replacement. These pores in the samples with cenosphere aggregate replacement were the reason for a reduction in the weight as compared to the control samples.

**Figure 16:**
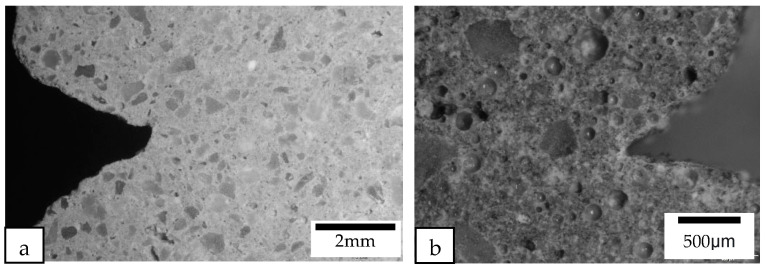
Macrostructure of the cross sections at the interlayer corner of (**a**) the printed control samples and (**b**) the printed samples containing cenosphere aggregate replacement were examined under a microscope.

All of the printed samples demonstrate a lack of obvious inter-bonding gaps between the layers, as shown by the edges of the different printed samples in Figure 16. The location of the bond interface between the layers is marked by these edges. The absence of gaps between the interface of the layers indicates that the cementitious material mixture containing different lightweight aggregates has high flowability characteristics, enabling the material near the interface to interact effectively during extrusion. Additionally, the short time gap between the layers allows the interfacing surfaces to interact producing a good bonding between the layers.

## 4. Conclusions

In this study, we adopt a systematic approach using different methods for formulating mixtures to improve the printing and mechanical performances of the different mixtures. Furthermore, the use of statistical optimization to determine the optimal percentage of each lightweight aggregate replacement and the improvement from each progression of the mixture formulation method proves the effectiveness of such an approach. During the initial phase of the study, only the specific gravity of the mixture was considered when formulating the mixture with different lightweight aggregate replacements. The test samples were analyzed to understand their material characteristics. Ensuring consistency was a challenge as the different lightweight aggregates used in the different mixture designs have very different material characteristics, which could cause the mixture to have high stiffness, and therefore, it could not be used for comparison. For example, as described in Section 3.1, the mixture design using perlite aggregate replacement resulted in a mixture that was too dry as compared to the control mixture design.

Additionally, we investigated packing factor and absorptivity to improve the flow characteristic of the different lightweight aggregate replacements. Amendments were made to improve the mixture design, as described in Section 3.2, where the different concrete mixtures obtained were compared and analyzed. The rheological analysis showed that using both the specific gravity and packing factor methods to develop the mixtures significantly improved the flow behavior of the mixtures. To optimize the mixtures’ performances, a statistical tool was used to maximize the specific compressive strength ratio, slump flow value, and static yield stress while minimizing the slump value, dynamic yield stress, and plastic viscosity. This was to ensure that a mixture had a high specific compressive strength, high flow characteristic, and good shape retention capability. However, the results are limited to the equipment and parameters used, and changes to the control mixture, nozzle size, or pumping mechanism could yield different results. Nonetheless, this study validated the mechanical capabilities and feasibility of printing optimized test samples with different lightweight aggregate replacements for concrete 3D printing applications. Based on the design objectives, it was determined that the optimal percentages of the cenosphere, perlite, and EPS foam bead aggregate replacements were 42%, 68%, and 44%, respectively. Finally, the optimized outcomes indicated that mixtures with cenosphere aggregate replacement exhibited the highest specific strength.

## Figures and Tables

**Table 1:** Printing parameters used for the experiment.

Nozzle Size	Travel Speed	Flow Rate
25 mm × 15 mm	100 mm/s	43 mL/s

**Table 2:** Percentage of absorption of different raw materials.

	River Sand	Perlite	Cenospheres	Foam Beads
Absorption (%)	0.596	49.286	3.078	4.167

**Table 3:** Packing factor values of the aggregates.

Aggregates	Packing Factor
Sand	0.66
Cenosphere	0.64
Perlite	0.14
Foam Beads (EPS)	0.56

## Data Availability

All available data presented in this study are contained within the article.
